# Combining transcriptional datasets using the generalized singular value decomposition

**DOI:** 10.1186/1471-2105-9-335

**Published:** 2008-08-08

**Authors:** Andreas W Schreiber, Neil J Shirley, Rachel A Burton, Geoffrey B Fincher

**Affiliations:** 1 Australian Centre for Plant Functional Genomics, School of Agriculture and Wine, University of Adelaide, Waite Campus, Glen Osmond, SA 5064, Australia

## Abstract

**Background:**

Both microarrays and quantitative real-time PCR are convenient tools for studying the transcriptional levels of genes. The former is preferable for large scale studies while the latter is a more targeted technique. Because of platform-dependent systematic effects, simple comparisons or merging of datasets obtained by these technologies are difficult, even though they may often be desirable. These difficulties are exacerbated if there is only partial overlap between the experimental conditions and genes probed in the two datasets.

**Results:**

We show here that the generalized singular value decomposition provides a practical tool for merging a small, targeted dataset obtained by quantitative real-time PCR of specific genes with a much larger microarray dataset. The technique permits, for the first time, the identification of genes present in only one dataset co-expressed with a target gene present exclusively in the other dataset, even when experimental conditions for the two datasets are not identical. With the rapidly increasing number of publically available large scale microarray datasets the latter is frequently the case. The method enables us to discover putative candidate genes involved in the biosynthesis of the (1,3;1,4)-*β*-D-glucan polysaccharide found in plant cell walls.

**Conclusion:**

We show that the generalized singular value decomposition provides a viable tool for a combined analysis of two gene expression datasets with only partial overlap of both gene sets and experimental conditions. We illustrate how the decomposition can be optimized self-consistently by using a judicious choice of genes to define it. The ability of the technique to seamlessly define a concept of "co-expression" across both datasets provides an avenue for meaningful data integration. We believe that it will prove to be particularly useful for exploiting large, publicly available, microarray datasets for species with unsequenced genomes by complementing them with more limited in-house expression measurements.

## Background

### Historical background

Measurements and comparisons of transcriptional activities of genes provide important information on the biological state of a cell. For example, enhanced transcription of a gene of unknown function in response to an imposed stress may be used to infer a possible biological function for the gene or, conversely, altered activity of a gene of known function may serve as a useful diagnostic indicator of the biological state. Several methods for measuring transcriptional activities of genes are in common use, such as those based on two-colour [1] and genechip [2,3] microarrays, serial analysis of gene expression [4], MPSS and other sequencing technologies [5,6] or on the real-time quantitative polymerase chain reaction (Q-PCR) [7]. Frequently, complementary data from several of these technologies are available for a particular biological system or process. This raises the question of how to perform a meaningful comparison and/or integration of transcriptional datasets from multiple sources.

Numerous approaches to transcriptomic data integration have been developed in recent years. For example, if the data originates from several sources using the same or similar platforms, a direct integration of expression values may be feasible [8,9]. For genes in common among the individual studies this can lead to increased significance of results upon integration simply by virtue of greater statistics. At the other extreme, if the platforms are dissimilar (e.g. two-colour cDNA and one-channel oligonucleotide arrays) then simple comparisons of expression values become meaningless. In this case a 'meta-analysis' of summary statistics such as fold-changes, p-values, ranks or effect sizes rather than expression values is more appropriate [10-16]. Choi [11,13] used this type of approach to compare two tumour-datasets, explicitly taking into account interstudy variation. Subsequently this approach was developed further in order to move beyond gene-by-gene analysis by constructing co-expression networks [17]. In a comprehensive work, Rhodes *et al*. used data from up to 40 published studies to identify common transcriptional signatures in diverse cancer microarray datasets [18,19]. More recently, Bayesian approaches for estimating model parameters within comparative analyses have been proposed (see, for example, [12,20] and references therein).

For the most part the above studies are concerned with improved diagnostic power through the integration of data, for example by decreasing p-values indicating differential expression of sets of genes desired as prognostic signatures for the detection of cancer. We are interested in quite a different line of inquiry, namely discovery of gene-function through co-expression across datasets. So, on the one hand we would like to work directly with expression values but, on the other hand, the expression data of interest are obtained from two very diverse platforms, in our case an Affymetrix array and a Q-PCR tissue set. Apart from obvious differences on the experimental side, the datasets obtained from these two platforms are themselves quite heterogeneous. In contrast to most integrative microarray studies, there is only a small overlap in gene content between our two datasets with only a few genes in common: a microarray dataset usually contains expression information for 10^3^–10^5 ^genes while a Q-PCR dataset typically consists of corresponding information for at most a hundred genes. As described in detail below, various other aspects of these datasets conspire to complicate a combined analysis even further. We do note, however, that both datasets correspond to measurements of absolute rather than relative gene expression.

### Experimental background

The organism we consider is barley (*Hordeum vulgare *L), where transcriptional information is often used to guide hypotheses about gene function and cellular processes because the regulation and function of only a small fraction of its genome is understood. The microarray data for this species, obtained with Affymetrix's Barley1 chip [21], is available through the barley reference experiment [22] (the data itself can be found at PlexDB [23]), which covers 15 different tissues and developmental stages. This dataset contains data from two barley cultivars; we make use of only one of these, namely 'Morex'. The barley microarray dataset is potentially useful for gene discovery because of a total of approx. 21400 non-redundant probesets on the Barley1 chip, the function of about 16500 genes cannot be reliably surmised from sequence comparisons with genes of known function in other species. However, it is a 'closed' dataset in the sense that the genes interrogated by the chip comprise a fixed fraction, perhaps half, of the genes in the genome [21] and this selection of genes is determined at the time of the design of the chip. Furthermore, because the probes on the chip were mostly designed from information available in the public EST databases, there is an inbuilt bias toward genes expressed at a significant level in at least one tissue, while genes transcribed at low levels are often missing from the chip.

**Figure 1:**
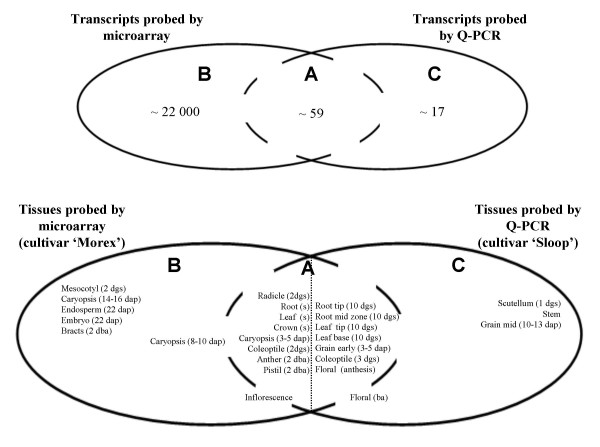
**The microarray and Q-PCR datasets**. The potential overlap of the microarray and Q-PCR datasets is depicted here. 'Region A' generically refers to the overlap between the datasets, 'Region B' to the part unique to the microarray and 'Region C' to that part unique to the Q-PCR series. It is clear from the context whether references to these regions in the main text refer to genes (top panel) or tissues probed (bottom panel). While up to 59 genes are simultaneously probed by both platforms, one would not necessarily expect their expression profiles to be identical in every case: differential contributions from (unknown) paralogs and/or closely related gene family members, as well as alternative splicing, can lead to distortions because the probes on the microarray and the primers used for the Q-PCR generally target different regions of a gene. Similarly, some tissues are probed simultaneously using both platforms, while others can be found in only one of the datasets. For a few tissues the overlap is hard to determine due to possible differences in developmental stage (shown in brackets; dgs = day old germinating seedling, dap = days after pollination, dba = days before anthesis, s = 10 cm seedling, ba = before anthesis). Further details about the tissues probed with the microarray may be found in Ref. [22], while details about those included in the Q-PCR series can be found in Ref. [48].

The Q-PCR based dataset, on the other hand, was taken from a series of 11 barley tissues, from the cultivar 'Sloop'. This dataset contains expression data for almost 80 genes that are mostly related to the synthesis or modification of cell wall polysaccharides. A number of these genes are only transcribed at relatively low levels and so it is not surprising that, while some of them are represented on the Barley1 chip, quite a few are unique to the Q-PCR dataset. The Q-PCR technique is more suited for detailed, targeted studies of genes of particular interest and, in contrast to the microarray dataset, it can be considered to be an 'open' dataset: it can easily be enlarged through the design of additional primers. For details on this Q-PCR data we refer to the Methods section as well as to the additional material [see Additional file 1]. The relationship between the genes and tissues probed in the two datasets is summarized in Figure 1.

### The experimental question

The central question that we would like to address here is the following: suppose one has identified a gene of interest that participates in a particular biological process and that one has collected Q-PCR data for the gene, but it is not a member of the set of genes represented on the microarray. For the reasons discussed above, this is frequently the case for species, such as most plant species, whose full genome has not been sequenced. We want to discover potential candidate genes involved in the same biological process as this gene of interest through a co-expression analysis, however a Q-PCR dataset consists, by its very nature, of expression data for only a very limited number of genes, so the value of carrying out this sort of analysis for this dataset alone is rather limited. On the other hand, microarray datasets contain expression information for a very large number of genes and would be ideally suited for the task.

For this reason we would like to use the extensive transcript data from the microarray to discover potential candidate genes involved in the same biological process as the original gene of interest. Co-transcription of these other genes identified from the microarray can then be verified in follow-up Q-PCR experiments. The stumbling block is, of course, that one needs to make a meaningful comparison between the actual expression profiles obtained from one dataset with those of the other. The difficulties with this include the following:

a) Each platform has inherent systematic errors that result in measurements that, while presumably correlated in some way to the actual mRNA concentrations, are distorted representations thereof. Intensive studies of this issue have been undertaken over the years, particularly between various microarray platforms [24-30]. It is fair to say that no clear consensus has emerged [31], with correlations between platforms ranging from 'poor' [26] to 'strong' [28]. Analogously, comparisons of fold-changes between microarray and Q-PCR measurements have demonstrated similar systematic, platform-dependent biases [32].

It appears likely that a significant source of differences across platforms is simply due to differential hybridization to platform-specific probes. This can arise because in general the probes on each platform can be expected to be sensitive to their own particular admixtures of alternative splice forms [33-36] and/or gene family members with similar sequence [35]. If a complete genome is available these admixtures can be identified, at least in principle, facilitating correct matching of probes between platforms [33,36]. For barley, however, the complete complement of genes, let alone splice forms, is not known.

b) The datasets considered here were obtained not only from different biological samples but also from different varieties of the same plant species.

c) The experimental conditions used to obtain one dataset correspond only partially with the experimental conditions used in obtaining the second dataset (see Fig. 1). Some of the experimental conditions are unique to one dataset, some to the other and some are common to both. Specifically, the experimental variables here consist of an assortment of tissues, some of which are unique to the genechip data (e.g. mesocotyl and embryo), some unique to the Q-PCR tissue series (e.g. scutellum and stem) and some are found in both datasets (e.g. caryopsis and root), although even in the latter case the age of the tissues probed is different.

d) As described above, the gene content of the two datasets is only partially overlapping and, in contrast to comparative microarray studies, highly asymmetric in size.

### The computational task

In short, our aim is twofold:

a) we want to establish a meaningful framework for quantifying the similarities and differences in the overlap of the two datasets (region A, Fig. 1) and

b) we want to use this mathematical framework to draw inferences about the non-overlapping parts of the datasets. In particular, we want to identify candidate genes probed *only *by the microarray (i.e. genes in region B) that are 'co-expressed' with genes probed *only *in the Q-PCR dataset (i.e. genes in region C).

### The Generalized Singular Value Decomposition

At first sight, the latter aim in particular poses a formidable, even impossible, challenge and, indeed, we are unaware of any existing methodology that would be suitable for realizing this goal. However, the datasets *do *contain some overlapping information (region A) and in this paper we show how to exploit this fact to achieve both tasks by using the matrix decomposition known as the generalized singular value decomposition (GSVD) [37-39].

The situation described is somewhat analogous to one that arises in the comparison of transcriptomes from two species [40]. In that case, certain genes rather than experimental conditions may be involved in processes common to the two organisms, while others may be involved in processes unique to either one or the other organism. The number of genes probed in the one species is in general different to that probed in the other and, of course, the ubiquitous systematic artefacts are present here as well. The latter problem has been addressed, using the GSVD, by Alter *et al*. [40] in a comparison of the cell cycle in humans and yeast. While here we are dealing with the orthogonal problem, it does have some similarity to the one considered in the pioneering study of Alter *et al*. and so a number of the conceptual ideas of the approach have already been introduced [40]. Here we concentrate on additional developments essential for the application of this novel matrix decomposition in its present setting.

More recently, Berger *et al*. [41] have used the GSVD approach to combine transcriptomic and copy number data obtained from genome-wide breast cancer studies. The aim in that work was similar to ours in that these authors set out to combine datasets collected from the same species from different experimental platforms. However, their approach is equivalent to that of Alter *et al*. in that the link between the two datasets was provided through coinciding experimental conditions – namely, identical time points in the cell cycle in Ref. [40] and identical cell lines in Ref. [41]. Because, as discussed above, the merging of transcriptomic datasets in general involves differing experimental conditions in the two datasets, the approach of Alter *et al*. and Berger *et al*. does not suffice for the problem addressed here. In addition, for the identification of candidate genes, we want to extend the use of the GSVD beyond a simple comparison of expression profiles of genes in common to the two datasets. In what follows we describe how to modify the approach to meet the more complex requirements of the present setting.

## Results

In this section we describe the application of the GSVD approach to a comparison of the two transcript datasets. The discussion is restricted to issues arising in a comparison of microarray and Q-PCR data, although it is straightforward to generalize it to other applications such as comparisons between genechip and two-colour arrays. The method is subsequently tested by comparing transcriptional profiles of genes common to the two datasets. Finally, the technique is applied by searching the microarray dataset for co-ordinately expressed genes for which only Q-PCR data are available. This leads to a testable biological hypothesis, implicating a particular glycosyl transferase in cell wall biosynthesis.

### Algorithm: Multiple gene-expression platforms and the generalized singular value decomposition

The well-known singular value decomposition of the *N *× *M *dimensional matrix *e*,

(1) *e *= *u *· *ε *· *v*^*T*^

has become a popular tool in the analysis of large-scale gene expression datasets [42] because it can be used to reorganize thousands of individual gene expression profiles, as measured by transcript abundance, into a small number of linearly independent processes involving linearly independent combinations of genes. The matrix *e *contains rows of 'gene-expression vectors' ***e***_***n***_, *1 *≤ *n *≤ *N*, with each component *e*_*nm *_indicating the level of transcription of gene *n *in array *m*, *1 *≤ *m *≤ *M*. In keeping with the nomenclature used by Alter *et al*. [42], we refer to the set of all gene expression values collected for a particular environmental condition as an 'array', even for the Q-PCR data; in our particular case one could, of course, simply refer to these as individual 'tissues'. As illustrated in the online Additional Material [see Additional file 2], it is useful to think of the *N *× *N *matrix *u *and the *M *× *M *matrix *v *as rotation matrices (hence *u*^*T*^·*u = I *and *v*^*T*^·*v = I*), rotating the original orthonormal coordinate systems spanned by individual genes and arrays to new coordinate systems [43]. Strictly speaking these matrices, being orthogonal, may involve reflections as well as rotations. A reflection corresponds to a change in the handedness of the new coordinate system, but the handedness is immaterial within the context of the present discussion. In the following, therefore, we take it as understood that our use of the term 'rotations' may include reflections as well.

The matrix *ε *contains the expression patterns as viewed from the new, rotated, coordinate systems and by construction it is very simple in that only its diagonal entries are non-zero. Singular value decompositions may of course be carried out individually for two datasets (labelled p and q), i.e. *e*^(*p*) ^= *u*^(*p*)^·*ε*^(*p*)^·*v*^(*p*)*T *^and *e*^(*q*) ^= *u*^(*q*)^·*ε*^(*q*)^·*v*^(*q*)*T*^, where the expression of the same set of genes has been measured in different sets of experiments. However, it is not possible to subsequently compare the expression matrices *ε*^(*p*) ^and *ε*^(*q*) ^directly, because the separate rotations *u*^(*p*) ^and *u*^(*q*) ^of the coordinate systems spanned by the genes have removed the information that there is a connection between genes in the two experiments. A simultaneous diagonalization may be achieved, however, through the use of the GSVD, defined by

(2) *e*^(*i*) ^= *y*·*ε*^(*i*) ^*·v*^(*i*)*T*^*   i *= *p*, *q*

The *N *× *N *dimensional matrix *y *again parameterizes the connection between the original and transformed genes, termed 'genelets' by Alter *et al*. [40], but now it is the same for both decompositions *p *and *q*. While this retains the desired common coordinate system for the genelet space, the price to pay is that the new 'genelet' axes are no longer orthogonal, that is, the matrix *y *is no longer purely a rotation/reflection matrix (hence *y*^*T*^·*y *≠ *I*). This geometrical interpretation of the SVD and GSVD is illustrated in the mathematical appendix contained in the online Additional Material [see Additional file 2].

The *M*^(*i*) ^× *M*^(*i*) ^dimensional matrices *v*^(*i*) ^define rotations from spaces spanned by arrays to spaces spanned by 'arraylets'. These rotations are necessarily different in the two datasets because the sets of experimental variables (in our case, the individual tissues) are unique to each dataset. As before, the *N *× *M*^(*i*) ^dimensional matrices *ε*^(*i*) ^only have non-vanishing entries *ε*_*nm *_^(*i*) ^if *n *= *m*, so each genelet is only expressed in its corresponding arraylet. By convention the singular values *ε*_*nm *_^(*i*)^are positive, decrease with increasing *n *in *ε*^(*p*) ^and increase with increasing *n *in *ε*^(*q*) ^[39,40].

The GSVD defined by Eq. 2 should be contrasted to that used in [40]. In that work, the GSVD was defined through *e*^(*i*) ^= *u*^(*i*)^·*ε *^(*i*)^·*x*^ -1^, where *u*^(*i*)^were rotation matrices connecting the gene and genelet co-ordinate systems while the non-orthogonal *M *× *M *matrix *x *^-1 ^was the matrix connecting array and arraylet co-ordinate systems. The reason for the difference between this definition and our Equation (2) is clear: in [40], the connection between the two datasets is made through coinciding experimental conditions, i.e. time-points in the cell cycle, while in the present case the connection between the datasets is imposed through coinciding genes. Hence in the former case a common transformation *x*^-1 ^from arrays to arraylets was required, while in the latter a common transformation *y *from genes to genelets is appropriate. Notwithstanding these differences in detail, transposition of *e*^(*i*) ^allows the same algorithm employed in [40] to be used for performing the decomposition in Eq. 2. Furthermore, as in [40], we use the angles

(3) $$\theta_{k}=\tan^{-1}\frac{\varepsilon_{kk}^{\left(p\right)}}{\varepsilon_{kk}^{\left(q\right)}}-\frac{\pi }{4}$$

as a measure of the relative contribution of the k^th ^arraylet and genelets to the first and second dataset. Those arraylets for which this angle is close to zero characterize processes that are common to the two datasets, while those arraylets for which *θ*_*k *_is close to *π*/4 or -*π*/4 characterize processes exclusive to the first or second datasets, respectively. The crucial observation is, therefore, that a sensible comparison between the datasets that avoids platform dependent biases should involve only those processes for which *θ *_*k*_≈*0*.

The proof of the GSVD in [37,40], and the corresponding algorithms implementing this decomposition, relies on the inequality *N *≤ *min(M*^(*p*)^, *M*^(*q*)^). Given that particularly microarray datasets typically contain many more genes than arrays, i.e. *N *>> *M*^(*i*)^, this implies that only a subset of probed genes may be used in Eq. 2. This is in contrast to the decomposition used by Alter *et al*. [40], where the inequality required the number of genes to be larger than the number of arrays, which is usually the case. While in principle the GSVD can be generalized to arbitrary *N*, *M*^(*p*) ^and *M*^(*q*) ^[38], only those genes in common between the two datasets (region A of Fig. 1) are represented in Eq. 2. Because part of our aim is to make use of gene expression profiles contained in only one or the other of the two datasets (regions B & C), we require further conceptual extensions to the analysis carried out in [40].

### The definition of the subspace in common to both datasets

The GSVD retains its utility in spite of these complications because it provides the transformations from the 'array'-space to 'arraylet'-space, i.e. *v*^(*p*) ^and *v*^(*q*)^, and at the same time identifies arraylets, for which *θ*_*k*_≈*0*, spanning the subspace of relevance to a comparison between the two datasets. It, therefore, provides a mathematical mapping from expression profiles in two disparate spaces, spanned by arrays, to a common space, spanned by arraylets. Ultimately, it is this feature of the GSVD which allows one make comparisons of expression profiles for genes contained in only one or the other datasets (i.e. genes in regions B and C).

The transformation between arrays and arraylets needs to be defined through the use of a suitable subset of genes common to both datasets (i.e. from region A; we shall refer to these as 'gene-pairs'). This subset defines arraylets characterizing common processes in the two datasets, relevant to this subset of genes. If one tabulates the expression of the *complete *set of genes in both datasets in the matrices *e*^(*p*) ^_*full *_and *e*^(*q*) ^_*full*_, one may write the expression of all genes (i.e. regions A, B and C) in the arraylets defined by the GSVD as

(4) $$\begin{array}{cc} y_{full}^{\left(i\right)}=e_{full}^{\left(i\right)}\cdot v^{\left(i\right)}.{\left(\varepsilon^{\left(i\right)}\right)}^{-1} & i=p,q \end{array}$$

Here (*ε*^(*i*)^)^-1 ^is the pseudo-inverse of *ε*^(*i*) ^[39]. This equation is the key result that we use in the present study.

The matrices ***y***^(*i*)^_*n*, *full *_contain the expression profiles of all genes in the two datasets. Each column contains the expression information for a particular arraylet and the relative contribution the arraylet *k *receives from each dataset is characterized by its angle *θ*_*k *_. Expression profiles of different genes (rows) in ***y***^(*i*) ^_*n*, *full *_may be directly compared, irrespective of whether they originate from regions A, B or C in Fig. 1.

Those genes actually used to define the GSVD will have identical expression profiles in the matrices *y*^(*i*)^_*full*_, i.e., ***y***^(*p*)^_*n*, *full *_= ***y***^(*q*)^_*n*, *full*_. Those genes contained in both datasets but not used to define the GSVD should have similar, but generally not identical, expression profiles ***y***^(*p*)^_*n*, *full *_and ***y***^(*q*)^_*n*, *full *_in the subspace (i.e. columns) characterized by *θ*_*k*_≈*0*. The degree to which these expression profiles correlate within this space provides a convenient measure of the utility of the GSVD and the suitability of those genes used to define it. Finally, expression profiles in the subspace characterized by *θ *_*k*_≈*0 *for genes present in only one or the other dataset alone (regions B and C) can also be compared, allowing the identification of putatively co-regulated genes.

**Figure 2:**
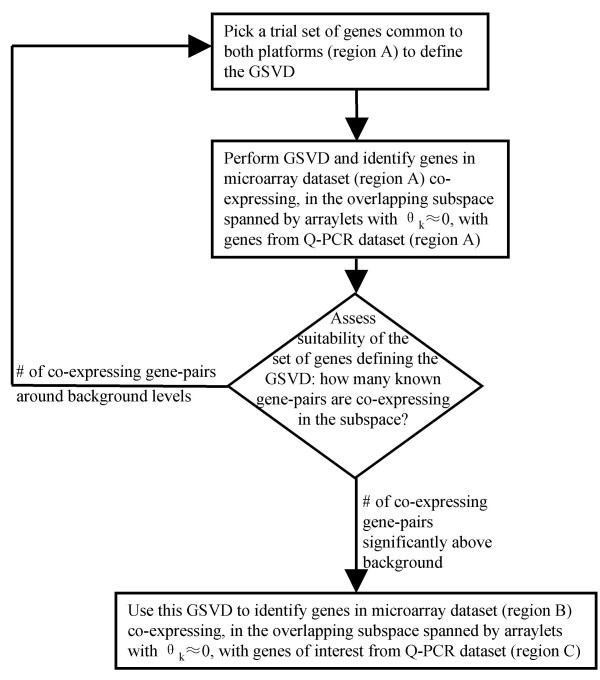
**Using the GSVD to identify candidate co-expressing genes**. This schematic flowchart shows the procedures used to identify a) an overlapping region between the two datasets as well as b) candidate genes probed by the microarray co-expressing with genes of interest from the Q-PCR dataset. Regions A, B and C refer to those defined in Fig. 1. In order to reduce the number of false positives we have repeated the entire procedure a number of times and only examine in detail genes that co-express consistently among these repeats.

These features suggest an iterative approach, illustrated in Fig. 2, for using the GSVD in a search for co-expressed genes across the two datasets. This approach is described in detail in the following sections.

### Testing the GSVD defined by random subsets of genes

We begin by illustrating the procedure using, at this stage, a *random *selection of gene-pairs from the overlapping region A in Figure 1 to define a GSVD of our microarray and Q-PCR data. The purpose here is twofold: firstly, we want to check that, as one would expect from the preceding discussion, the expression profiles of the remaining gene-pairs from region A indeed show greater co-expression in the subspace spanned by the central arraylets than those spanned by peripheral arraylets. Secondly, this illustration provides a vehicle for introducing the particular measure that we shall adopt for quantifying "co-expression". This measure will be used in the subsequent analysis.

We use a random selection of 10 gene-pairs in the definition of the GSVD. The size of this set is dictated by the requirement *N *≤ *min(M*^(*p*)^, *M*^(*q*)^) discussed above, i.e. in our case we need *N *≤ 11. We have used one gene-pair less than this because, for convenience, the datasets have been standardized by centering each gene's transcription profile and scaling its variance across the tissues to unity. The centering results in one column in each matrix becoming linearly dependent on the other 10. Finally, in keeping with standard practice, we have worked with the *log*_2 _of the expression intensities.

**Figure 3:**
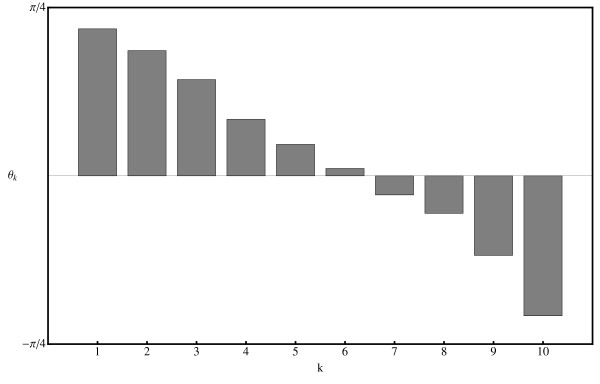
**Relative contribution of arraylets/genelets to the two datasets**. Genelets with *θ*_*k *_> 0 (i.e. small k) correspond to those expressed predominantly in the microarray dataset while genelets with *θ*_*k *_< 0 (i.e. large *k*) correspond to those expressed predominantly in the Q-PCR dataset. The angles *θ*_*k *_result from a GSVD defined by the genes marked by asterisks in the Table in the online Additional Material [see Additional file 1].

The random set of 10 genes used for the GSVD is indicated by asterisks in the table available in the online Additional Material [see Additional file 1]. The resulting range of angles *θ *_*k *_is shown in Fig. 3. It is evident from this figure that arraylets *k *= 5, *k *= 6 and *k *= 7 contribute a similar amount to both datasets, while arraylets *k *≤ *4 *increasingly dominate in the microarray dataset and arraylets *k *≥ *8 *increasingly dominate in the Q-PCR dataset. One would expect, therefore, to have the greatest success in making an identification of genes between the two datasets if the overlapping subspace included the central arraylets *k *= 5, *k *= 6 and *k *= 7.

There is of course arbitrariness in how one actually defines the "identification of genes". A convenient procedure adopted here consists of calculating, for each microarray gene $M_{p}$ in turn [see Additional File 1], the Euclidean distance *d *within the central arraylets for all Q-PCR gene transcripts ($Q_{q}$), i.e. for each *p *we calculated *d*_*c*.*a*._($M_{p}$, $Q_{q}$) for all *q*. We chose to define a "successful match" to be one where the appropriate gene from the Q-PCR dataset (*p *= *q*) is one of the seven 'closest' to the microarray gene, i.e. rank(*d*_*c*.*a*._($M_{p}$, $Q_{q}$) ≤ *7*). While the absolute number of "successes" is naturally sensitive to this arbitrary choice of the cut-off, comparisons between them are less so.

The results from this illustrative exercise are shown in Table 1. In this instance the greatest success is achieved using either arraylets 4 to 8, 5 to 9 or 3 to 9. In these cases 17 out of 49 genes are successfully matched. The success rate decreases, as one would expect, if non-central arraylets are chosen. For example, using the Q-PCR dominated arraylets 8–10, only 10 genes are successfully matched. Similarly, using the microarray dominated arraylets 1–3 only 4 genes are matched. Successful matches may of course occur purely by chance, with a binomial probability distribution given by *Pr*(*j*, *J*; *x *= *s*/*S*) = *J*!/[*j*! (*J*-*j*)!] *x*^*j*^(1-*x*)^*J*-*j*^, where *Pr*(*j*, *J*; *x*) is the probability of having *j *successes in *J *= 49 trials by randomly picking *s *= 7 genes from a list of *S *= 59. The p-values associated with this null-hypothesis are shown in brackets in Table 1. The rate of success achieved by matching gene expression in the central arraylets of the GSVD is clearly far greater than one would expect by chance, with typical p-values in the range of 10^-5 ^to 10^-3^. This success rate decreases, as expected, to around background levels (i.e. p-value of order 1) in the peripheral arraylets.

**Table 1:** The number of correctly identified genes as a function of both subspace location and dimension

k	Δk = 0	Δk = 1	Δk = 2	Δk = 3	Δk = 4
1	4 (0.85)				
2	6 (0.53)	4 (0.85)			
3	5 (0.71)	6 (0.53)	5 (0.71)		
4	3 (0.94)	6 (0.53)	10 (5.9 × 10^-2^)	5 (0.71)	
5	8 (0.22)	14 (1.3 × 10^-3^)	13 (3.8 × 10^-3^)	13 (3.8 × 10^-3^)	8 (0.22)
6	12 (1.1 × 10^-2^)	13 (3.8 × 10^-3^)	17 (2.7 × 10^-5^)	17 (2.7 × 10^-5^)	5 (0.71)
7	9 (0.12)	14 (1.3 × 10^-3^)	17 (2.7 × 10^-5^)	14 (1.3 × 10^-3^)	
8	9 (0.12)	15 (3.9 × 10^-4^)	13 (3.8 × 10^-3^)		
9	8 (0.22)	10 (5.9 × 10^-2^)			
10	8 (0.22)				

Arraylets ranging from *k-Δk *to *k+Δk *were used to define the subspace. The genes used to define the GSVD are indicated by asterisks in the online Additional Material [see Additional file 1]. The numbers in brackets are the corresponding p-values.

**Figure 4:**
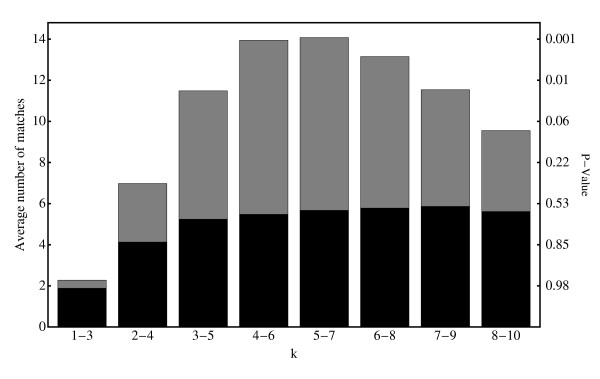
**The *average *number of successfully identified microarray genes, using distance within three arraylets as the measure of similarity (light bars)**. On the right hand axis the calculated p-values characterizing the expected number of false positives for a single GSVD are shown. The dark bars indicate the result obtained if those microarray genes not used in the GSVD are randomized.

Naturally, the results shown in Table 1 depend on the particular set of gene-pairs used to define the GSVD and, indeed, considerable fluctuations around these numbers may be observed when choosing a different set of genes to define the GSVD. In view of this one may well ask to what extent the results in Table 1 are 'typical'. We have investigated this by using the fact that random fluctuations may be averaged out by performing large numbers of GSVD's, defined by randomly chosen sets of 10 gene-pairs. The results of a series of 1000 GSVDs defined in this way are shown in Fig. 4, indicating that the general trends observed in Table 1 are robust: The greatest success in matching Q-PCR and microarray profiles is achieved in the central arraylets with an *average *of around 14 positive matches. Also shown in Fig. 4 are the p-values associated with the null-hypothesis for a single GSVD (see Table 1) as well as, shaded dark, an additional check that the results are not being over-interpreted. Here the expression profiles in the microarray data not used in defining the GSVD were randomized, thus destroying all remaining inherent biological connections between the two datasets, before performing the matching procedure. Similar results are obtained if the microarray expression profiles are randomized before defining the GSVDs (data not shown). For all but the smallest *k *these results are consistent with the average expected background, *Js/S ≈ 5.81*.

We conclude, therefore, that co-expression of known gene-pairs is indeed strongest, and highly significant (p-value < 10^-3^), in the subspace spanned by the central arraylets. On the other hand, in peripheral arraylets co-expression of known gene-pairs occurs at background levels. This provides strong empirical evidence that search for co-expression in the subspace spanned by the central arraylets indeed provides a tool for identifying candidates for co-expressed genes across the two datasets.

### Improving the GSVD through a judicious choice of defining gene-pairs

While the discussion so far addresses the utility of the GSVD in dealing with partially overlapping experimental conditions in the two datasets, we shall now address the second problem illustrated in Figure 1: because the primers used for the Q-PCR target different regions to the probes on the microarray, there is some uncertainty in defining the set of genes that are part of region A in the first place. It could well be that alternative splice forms, unknown paralogs and/or gene family members with closely related sequence contribute differently to the signal obtained with the two platforms. Clearly, it would not be wise to make use of cases like this in defining the common subspace between the two experiments.

We contend that the freedom one has in selecting the set of gene-pairs defining the GSVD allows one to test whether or not the set contains 'contamination' of this sort and that this freedom, therefore, provides a solution to this problem. In particular, it should be noted that although the results shown above are typical, some sets of gene-pairs improve the performance of the GSVD in matching genes in the two datasets dramatically. We have found over a dozen sets of *10 *gene-pairs that result in *35 *to *39 *out of *49 *successful matches. While one naturally expects some fluctuations in this success-rate it is easy to check, using the binomial distribution discussed earlier, that fluctuations of this magnitude and frequency go significantly beyond what one would expect by chance alone. Given the difficulties that have been encountered in previous cross-platform comparisons of gene expression data [24-31], this is a notable result. A natural interpretation of this success is that these sets of gene-pairs define GSVDs that are able to cope particularly well with systematic platform dependent artefacts, that the expression of these genes shows little or no cultivar dependence and that differential sensitivity to alternative splice forms etc. for these gene-pairs is not an issue.

A corollary of this line of reasoning is that expression signals of gene-pairs that are strongly affected by any of these artefacts, when used to define the GSVD, consistently lead to poor results. Indeed, this is found to be the case. For example, inclusion of the barley cellulose synthase-like gene *HvCslE2 *in the set of 10 genes used to define the GSVD invariably leads to a low number of successful matches. At the same time, direct comparison of expression profiles of *HvCslE2 *in both the microarray and Q-PCR tissue series indicates that, while on the microarray expression of this gene in caryopsis both 8–10 and 14–16 days after pollination is somewhat down-regulated as compared to the average across all tissues, in the Q-PCR dataset it is strongly up-regulated in the tissue that roughly corresponds to these, namely developing grain 10–13 days after pollination. While the origin of this apparent discrepancy is not known, it illustrates how one can gain information on the (in)-consistency of the expression profiles of individual gene-pairs in the two datasets by using the GSVD. In summary, we conclude that in addition to using subspaces defined only by central arraylets of the GSVD, one can further greatly improve the efficacy of the method by defining the GSVD using sets of genes that maximize its success rate for matching genes in common in the two datasets. This procedure is summarized in Fig. 2.

Various strategies for selecting suitable gene-pairs may be employed. In most cases an exhaustive brute force search for the optimum set is not feasible: in our case this would have entailed testing $\left(\begin{array}{c} 59\\ 10 \end{array}\right)$ ≈ 6 × 10^10 ^combinations of gene-pairs. As an alternative heuristic method one may start with a random set of gene-pairs and progressively swap new gene-pairs from region A into this set, keeping those that lead to improved gene-pair matching. We elected to implement a combination of these approaches: first, we narrowed down the choice of suitable gene-pairs to 20 through a heuristic search for gene-pairs that tended to improve performance and then exhaustively tested all selections of length 10 (i.e. $\left(\begin{array}{c} 20\\ 10 \end{array}\right)$ = 184,756 of them) picked from this narrowed down set.

## Discussion

### Implementation of the GSVD

Finally, we turn to applying the methodology developed in this paper to a real biological problem. We are interested in a particular gene for which Q-PCR expression data has been collected but for which microarray information is not available (i.e. a gene in region C of Figure 1). This gene is a member of a barley cellulose synthase-like gene family and is designated *HvCslF3 *(GenBank Acc. No. EU267179; for details of the biological methods as well as the numerical results of the Q-PCR experiments, see the Methods section as well as the online Additional Material [see Additional file 1]). It has recently been implicated in the biosynthesis of the polysaccharide (1,3;1,4)-*β*-D-glucan, which is a major constituent of cell walls of commelinoid monocotyledons, including barley [44,45]. However, given the presence of two distinct linkage types and the general structural complexity of barley and other (1,3;1,4)-*β*-D-glucans, it might be anticipated that additional enzymes could be required for the biosynthesis of the polysaccharide and for its post-synthetic modification, either during transport to the cell wall or following its deposition into it [46,47]. For example, in cellulose biosynthesis, groups of at least three cellulose synthase enzymes (HvCesA's) are thought to be required for the formation of the active terminal rosette complex through which cellulose microfibres are secreted into the cell wall [48]. Furthermore, the mRNAs encoding the cellulose synthase-like HvCslF proteins are often of relatively low abundance [49] and corresponding gene sequences are generally under-represented in EST databases. As a result, only one representative of seven known members of the *HvCslF *gene family is found on the Barley1 microarray [48], despite the fact that the chip includes over 22,000 contigs [21]. Thus, Q-PCR data obtained for the *HvCslF3 *gene was combined with the microarray data, using the GSVD, to identify co-transcribed genes from the chip that might provide clues to the identities of ancillary proteins or enzymes required for (1,3;1,4)-*β*-D-glucan biosynthesis.

A GSVD analysis was performed using a particular set of 10 gene-pairs from region A in Figure 1 (*HvCesA1, HvCesA2, HvLimit-Dextrinase Inhibitor, HvCesA4, HvGlyT5, HvCesA8, HvUXS3, HvCslC4, HvEndogluII, HvGSL3*). This set was chosen because it resulted in a large number (39/49) of matches for the remaining gene-pairs in region A ("matches" being defined as gene-pairs sufficiently close in Euclidean distance in the subspace spanned by the central arraylets 5 to 7, as described in detail earlier on). Using Equation (4), this GSVD provides the mapping from the space spanned by arrays to the space spanned by arraylets for the remaining genes in regions B and C of Figure 1. Transcripts from the microarray (i.e. from region B) co-ordinately transcribed with the *HvCslF3 *gene (from region C), within the subspace spanned by the central arraylets, could then be identified.

**Figure 5:**
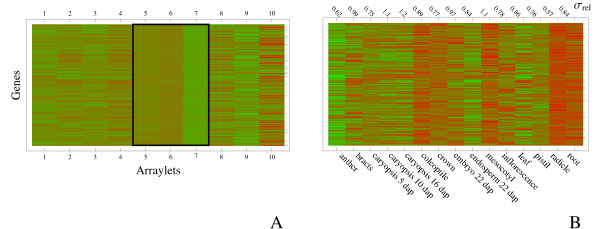
**Relation between co-expression within the central arraylets to co-expression in the microarray data**. Panel A shows gene expression as measured in the arraylets defined by the GSVD using a set of genes described in the main text (green – low expression, red – high expression). Only the 200 transcripts whose expression profile in the central arraylets 5–7 (boxed) is closest to that of *HvCslF3 *(as measured by Euclidean distance) are shown. The expression profiles for the same genes, in the space spanned by arrays, are shown in panel B. Approximate co-expression can be seen for some tissues (e.g. expression in anther, caryopsis and endosperm tends to be low, while expression in root, radicle and coloeptile tends to be higher). At the top of panel B we indicate, for each tissue, the standard deviation of expression values among the genes shown on the plot, scaled with the corresponding quantity for the whole dataset; i.e. values larger (smaller) than one indicate larger (smaller) variability than average. As expected (see text), variability in expression is smallest in those tissues represented in both datasets.

It is illustrative to compare this co-expression in the space spanned by arraylets to the expression profiles obtained directly from the microarray. In Fig. 5A we show a heatmap of 200 transcript abundances obtained with the microarray, ordered so that those co-expressing most closely with *HvCslF3 *in the central arraylets are at the top of the plot. The co-expression in the central arraylets is clearly visible. On the other hand, little or no co-expression in the peripheral arraylets characterising expression in non-overlapping parts of the datasets is apparent. For comparison, the corresponding expression profiles in the original space spanned by the arrays of the microarray experiment are shown in Fig. 5B. Some overall trends are apparent: expression in anther, caryopsis and endosperm tends to be low for these genes, while expression in root-like tissues and coleoptile tends to be high. More interesting, however, is the variation in expression among these genes. Co-expression in central arraylets should be reflected in stronger co-expression in tissues that are in common between the two platforms than those tissues that are not. As a measure of this variation we have listed, along the top of Fig. 5B, the standard deviation of expression among these 200 genes, scaled by the corresponding quantity for the whole dataset. We see that a selection of genes based on co-expression within central arraylets has resulted in a gene-set that is most tightly co-expressed in anther, caryopsis (5 dap), crown, inflorescence, pistil and radicle, but co-expressed less than average in caryopsis (8–10 & 14–16 dap) and mesocotyl. Comparing with Figure 1 we see that the former tissues are mostly those probed by both tissue series, while the latter are among those probed by the microarray alone. It appears therefore that, as expected, the central arraylets of the GSVD are indeed associated with those tissues for which there is some overlap between the two series.

Naturally, there are many 'co-expressed' genes and, in principle, one could perform follow-up analyses on all of those that are sufficiently co-expressed with *HvCslF3 *within the subspace under consideration. At this stage, we have focussed our attention only on those genes that, in addition to being co-expressed, are also already suspected to participate in cell wall synthesis on the basis of their annotation. A list of 20 of these genes, with the highest scores for co-expression with *HvCslF3*, is shown in Table 2.

**Table 2:** Microarray probesets with profiles closest to the Q-PCR profile for the cellulose synthase-like gene *HvCslF3*.

Barley1 probeset	Dist.	Annotation (E-value)
*Contig12242*	0.17	UDP-glucose:sterol Gt [As] (1 × 10^-67^)
*Contig14077*	0.40	putative glycosyltransferase [Hv]; (1 × 10^-160^)
*HVSMEa0015K08r2*	0.42	putative XTH [Os] (3 × 10^-13^)
*HV06O09u*	0.46	putative glucosyltransferase [Os] (1 × 10^-35^)
*Contig11619*	0.48	ceramide glucosyltransferase [Ga] (8 × 10^-51^)
*Contig6602*	0.52	putative glycoprotein 3-*α*-L Ft [Hv] (0)
*Contig14830*	0.55	putative glucosyltransferase [Os] (1 × 10^-113^)
*HE01I24u*	0.57	xyloglucan endo-1,4-*β*-D-Gl [Hv] (6 × 10^-27^)
*Contig11983*	0.64	galactosyltransferase family [At] (1 × 10^-113^)
*HV12D17u*	0.70	putative GDP-fucose protein-Ft [Os](2 × 10^-52^)
*Rbags19k14*	0.74	putative glucosyltransferase [Os] (2 × 10^-20^)
*Contig2958*	0.74	XTH [Hv] (1 × 10^-170^)
*Contig18221*	0.75	XYLT [At] (1 × 10^-111^)
*Contig23070*	0.76	GALT family-like protein [Os] (3 × 10^-70^)
*HVSMEl0008B06r2*	0.76	putative GT family [Os] (5 × 10^-28^)
*HVSMEl0013E16r2_s*	0.77	putative xyloglucan Ft [At] (8 × 10^-10^)
*Contig14826*	0.78	putative glucosyltransferase [Os] (6 × 10^-58^)
*Contig15434*	0.79	glycogenin GT [Os] (4 × 10^-53^)
*Contig15291*	0.80	putative glucosyltransferase [Os] (7 × 10^-48^)
*Contig5876*	0.81	putative glucosyl transferase [Os] (0)

The distance in the second column is the Euclidean distance in the subspace spanned by arraylets 5–7 of the GSVD defined by 10 genes in common between the two datasets (*HvCesA1, HvCesA2, HvLimit-Dextrinase Inhibitor, HvCesA4, HvGlyT5, HvCesA8, HvUXS3, HvCslC4, HvEndogluII, HvGSL3*).

Abreviations: As – Avena sativa; At -Arabidopsis thaliana; Ga – Gossypium arboretum; Hv – Hordeum vulgare; Os – Oryza sativa; Ft – fucosyltransferase; GALT – galactosyltransferase; Gl – glucanase; Gt – glucosyltransferase; XTH -xyloglucan endo-transglycosylase; XYLT – beta-(12)-xylosyltransferase.

Furthermore, one may wonder to what degree the results in Table 2 reflect the choice of 10 genes used to define the GSVD in the first place. Another choice for this set of genes may well lead to some changes to the list of putative co-expressors listed in Table 2. In other words, it may well be that subsequent analyses could expose some of these candidate genes as being false positives. In order to reduce this number of false positives we elected to repeat the procedure shown in Fig. 2 a number of times, each time using another set of 10 genes to define the GSVD. Each of these sets (16 in total) was chosen because it resulted in a similarly large number of matches of pairs of genes in common in the datasets as the first one. It was comforting to find that there are quite a number of genes in the lists of co-expressors that are insensitive to the choice of gene-pairs used to define the GSVD: we found that in *all *cases *Contig11619 *(annotated as a ceramide glucosyltransferase) is co-transcribed with *HvCslF3*, in 15 out of 16 GSVD analyses *Contig14830 *(annotated as a putative glucosyltransferase), *Contig15434 *(the cellulose-synthase-like gene *HvCslA4*) and *Contig18825 *(the cellulose-synthase-like gene *HvCslC1*) were co-expressed and, in 14 out of 16 analyses *Contig16931 *(annotated as a galactoside 2-*α*-L-fucosyltransferase), was co-expressed. While our subsequent analysis concentrated on these genes it could well be that other transcripts in Table 2 (or, for that matter, other transcripts not annotated as cell wall related) may also be worthy of further investigation.

### Confirmation of co-expression using Q-PCR

**Figure 6:**
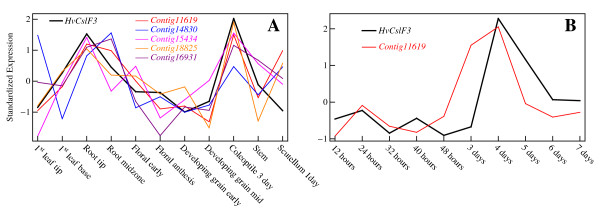
**Confirmation of co-expression of *HvCslF3 *and candidate genes**. In panel A the Q-PCR expression profiles of the cellulose synthase-like gene *HvCslF3 *and the candidate genes identified in this study are compared. Expression profiles have been standardized as described in the text. As can be seen, the genes indeed co-express in the tissues probed in both the Q-PCR and microarray datasets. Panel B shows an additional comparison of Q-PCR coleoptile time course expression profiles of *HvCslF3 *and *Contig11619*. The two genes appear to remain roughly co-expressed in this time-course as well.

In order to confirm the apparent co-regulation of *HvCslF3 *with this selection of genes probed by the microarray, primers were constructed so that their transcript abundance in the 11 barley tissues of the Q-PCR dataset could be checked directly using Q-PCR. The resulting expression profile of the most consistently co-expressed candidate (correlation coefficient 0.72), the putative ceramide glucosyltransferase *Contig11619*, is shown in red in Figure 6A alongside the corresponding expression profile of *HvCslF3 *(black), confirming that the GSVD procedure has indeed correctly identified a hitherto unknown co-expressed gene to this cellulose synthase-like gene. Similar cross-checks were carried out for *Contig14830 *(corr. coeff. 0.29), *Contig16931 *(0.68), *Contig15434 *(0.75) and *Contig18825 *(0.71), the latter two being already present in the Q-PCR dataset (i.e. region A). The expression profiles for these genes are also shown in Fig. 6A. As can be seen, all but *Contig14830 *show significant co-expression with *HvCslF3 *in the tissues probed by the Q-PCR dataset.

It is noteworthy that the co-expression of *HvCslF3 *with *Contig11619 *breaks down in scutellum. This is a tissue that is part of the Q-PCR dataset but not the microarray dataset. Quite correctly, therefore, the central arraylets that were searched for co-expressed genes were insensitive to the expression level in this tissue (the analogous behaviour for tissues probed in the microarray dataset but not the Q-PCR dataset has already been noted in Figure 5B). While the origin of the lack of co-expression is not known at present it should be noted that in a further series of Q-PCR based measurements, using coleoptiles at different stages of development (R. A. Burton, unpublished data), close coordinate transcription of the *HvCslF3 *and ceramide glucosyl transferase persisted (Figure 6B). Similarly, the apparent lack of co-expression of the Q-PCR derived profiles of *Contig14830 *and *HvCslF3 *is most noticeable in those tissues where the Q-PCR series indicates significant sub-tissue dependence (leaf-tip vs. leaf-base, root-tip vs. root midzone), sub-tissues that were not probed individually in the microarray experiment.

## Conclusion

In summary, we have applied the generalized singular value decomposition to the combined analysis of two expression datasets that are only partially overlapping in both gene content as well as experimental conditions. This adapts and significantly extends the use of the GSVD beyond its original use in gene expression analysis, namely a comparative study of cell cycles of two species where the experimental conditions were identical. The extension makes use of a selection procedure that adjusts the set of genes used to define the GSVD in order to maximize expression-profile matching of known gene-pairs in the two datasets. In this way, one effectively uses the information contained in the expression data itself (rather than probe-matching to a reference sequence) to eliminate gene-pairs whose expression signal may be affected by differential sensitivity to alternative splice forms and/or other gene family members. Furthermore, we have demonstrated that the resulting decomposition provides an effective framework for conducting searches for candidate genes in one dataset that are likely to co-express with genes contained only in the other dataset.

The methodology developed here has provided testable leads for the identification of genes that might be co-ordinately transcribed with the *HvCslF3 *gene. Indeed, the association of the most consistently co-expressed candidate, the ceramide glucosyl transferase, with *HvCslF3 *is quite plausible. It might form part of the cellular machinery necessary for the biosynthesis of cell wall (1,3;1,4)-*β*-D-glucans. Ceramide mono- and oligoglucosides are members of the glycosphingolipid group of plant plasma membrane components that are believed to separate laterally to form specialized microdomains in the membrane [50,51]. These so-called 'lipid rafts' are believed to recruit groups of proteins, including GPI-anchored proteins and integral membrane proteins, that assemble in localized areas for specialized membrane processes [52]. Furthermore, it has recently been shown that GPI-anchored proteins are required for cell wall biosynthesis and morphogenesis in Arabidopsis [53] and it has earlier been suggested that glycolipids or steryl glycosides might act as intermediates in the biosynthesis of wall polysaccharides [54].

The GSVD procedure described here has allowed the combination of Q-PCR and microarray transcript datasets and, through this integration, the development of testable hypotheses as to which genes might be involved in specific cellular processes. More generally, the procedure dramatically extends the utility of a limited dataset of Q-PCR analyses, for a small number of genes of interest, through combination with much larger microarray datasets. The GSVD analysis should be of similar value in combining other types of transcript datasets in any biological system for which microarray, MPSS or other large transcript datasets are available.

## Methods

### Real Time Quantitative-PCR

Barley tissues were prepared, RNA extracted and cDNA synthesized as detailed in Burton *et al*. [48]. The amount of cDNA required to perform the experiments described here meant that two aliquots of cDNAs were prepared and combined for all tissues, using the same RNA preparations.

Stock solutions of the PCR product for the preparation of a dilution series were prepared from the cDNAs and purified and quantified by HPLC [48]. A dilution series covering seven orders of magnitude was prepared from 10^9 ^copies/*μ*l stock solution [48]. Three replicates each of seven standard concentrations were included with every Q-PCR experiment together with a minimum of three 'no template' controls. Some Q-PCR experiments were assembled by hand and others were assembled using a CAS-1200 liquid handling robot. Three replicate PCRs for each of the cDNAs were included in every analysis.

Reactions were performed in an RG 3000 Rotor-Gene Real Time Thermal Cycler as follows; 15 min at 95°C followed by 45 cycles of 20 s at 95°C, 30 s at 55°C, 30 s at 72°C and 15 s at an optimized acquisition temperature. A melt curve was obtained for the final product by heating from 70°C to 99°C. The optimal cycle threshold (CT) was determined from the dilution series using the Rotor-Gene V6 software, and the raw expression data were derived. The mean expression levels and standard deviations for each set of four replicates for each cDNA were calculated and were normalized using the procedure described in Burton *et al*. [48].

### Microarray Data

Sequences for the Q-PCR products of all primers used in this study were made available to us by members of our laboratory. These sequences were compared with the Affymetrix Barley1 genechip sequences using the Blast-n algorithm [55]. Matched sequences were defined by demanding an E-value better than 10^-38 ^and a percent-identity better than 93%. The precise value of these cut-offs is not crucial: increasing the stringency to E-value < 10^-50 ^and P.I. > 95% eliminates only 2 matched sequence pairs. In a small number of cases the matching was ambiguous in that several different genechip sequences with similar E-values were found. These cases were not included in the matched set. The results are summarized in the online Additional Material [see Additional file 1].

## Authors' contributions

AWS and NJS jointly conceived the methodology described in this paper. AWS carried out the GSVD calculations and drafted the manuscript. NJS and RAB created the Q-PCR dataset. GBF provided the motivation for this work and was involved in drafting the manuscript. All authors read and approved the final manuscript.

## Supplementary Material

Additional file 1 Table S1. This Excel spreadsheet contains Table S1 with the Q-PCR data used in the paper. Click here for file

Additional file 2 The geometrical interpretations of the singular and generalized singular value decompositions. This Word document contrasts the geometric interpretations of the singular and generalized singular value decompositions. Click here for file
