# Supplementary material for: Combining transcriptional datasets using the generalized singular value decomposition
Source: BMC Bioinformatics. 2008 Aug 8;9:335. doi: 10.1186/1471-2105-9-335 (PMC2562393; doi:10.1186/1471-2105-9-335)
Supplement: Additional file 2 — The geometrical interpretations of the singular and generalized singular value decompositions. This Word document contrasts the geometric interpretations of the singular and generalized singular value decompositions. [file 1471-2105-9-335-S2.doc]

**The geometrical interpretations of the singular and generalized singular value decompositions.**

As indicated in the main part of this paper, when discussing the meaning of the singular and generalized singular value decompositions applied to gene expression data a geometrical interpretation is useful. In this appendix we illustrate this geometrical picture using a simple example of datasets consisting of only two genes whose expression has been measured in two tissues only.

The rows of the expression matrix *e*, decomposed in a SVD (Eq. 1) as

$e=u\cdot\varepsilon\cdot v^{T}$ (5)

consist of vectors ***e****_n_* whose components specify the expression level of the n^th^ gene in the individual arrays, as shown in Fig. S1 for a system of two genes & two arrays. The matrix *v* defines a new array-coordinate system, indicated in red in Fig. S1. In two dimensions this matrix can be parameterized by a single angle *θ_v_*. If det(*v*) = 1, the matrix is a pure rotation matrix while, if det(*v*) = -1, it also includes a reflection. For simplicity we deal here only with the former case, the latter merely serving to re-define the handedness of the new coordinate system. The components of the vectors ***e****_1,2_* along this rotated coordinate system specify the expression levels of the two genes in the first and second eigenarray, respectively. At the same time the columns of the matrix *v* define linear combinations of genes termed ‘eigengenes’. It is the particular feature of the SVD that in this new eigenarray-coordinate system each eigengene is only expressed in its corresponding eigenarray, i.e. in Fig. S1 the vector ***v****_1_* (***v****_2_*), shown as red arrows, falls on the first (second) axis of the rotated coordinate system. The complexity of the original expression matrix *e* has been moved into the connection between the old and new coordinate systems provided by *u* and *v*, while the expression matrix *ε* in the new coordinate systems is exceedingly simple.

Analogously, the columns of *e* may be thought of as vectors ***a****_m_* whose components specify the expression level of individual genes in the *m^th^* array, shown in Fig. S2. The rotation matrix *u* (parameterized in 2D by the angle *θ_u_*) defines a rotated gene-coordinate system, indicated in red. The components of the vectors ***a****_1,2_* along this rotated coordinate system specify the expression levels of individual genes in either array *1* or *2*. This time the columns of the matrix *u* also define linear combinations of arrays termed ‘eigenarrays’ and, consistent with above, the eigenarrays (red arrows) defined by the SVD only receive contributions from the corresponding eigengene.

The GSVD defined by

$\begin{matrix} e^{(p)}=y\cdot\varepsilon^{(p)}\cdot v^{(p) T} \\ e^{(q)}=y\cdot\varepsilon^{(q)}\cdot v^{(q) T} \end{matrix}$ (6)

may be thought of as individual rotations *v^(p)^* and *v^(q)^* of the coordinate systems defined by the arrays in datasets *p* and *q*, as well as a common transformation *y* (not a rotation!) from the coordinate system defined by the genes to one defined by ‘genelets’ [40]. The rotations from arrays to arraylets for the dataset *q* are shown in Fig. S3 and are analogous to those depicted in Fig. S1, with the angle $\theta_{v}^{(q)}$ defining the matrix *v^(q)^*, respectively. An equivalent plot (not shown) could be constructed for the dataset *p*.

On the other hand, because each gene makes a contribution to both the arrays in the datasets *p* and *q*, the array-expression vectors ***a*** and ***a*** may all be plotted in a single diagram corresponding to Fig. S2 - see Fig. S4. The ‘genelet coordinate system’ is no longer orthonormal, with the rotation of each axis (indicated by$\theta_{y}^{(n)}$) determined by the corresponding column in the matrix *y*. The contribution that the *m^th^* array of either dataset receives from the corresponding *n^th^* genelet is no longer given by a perpendicular projection of ***a****_m_* onto the *n^th^* eigengene's axis, as indicated by the dashed red lines in Fig. S4.

As with the singular value decomposition, the *N×M^(i)^* dimensional matrices *ε^(i)^* only have non-vanishing entries $\varepsilon_{nm}^{(i)}$ if *n=m*, so again each genelet is only expressed in its corresponding arraylet as indicated in Figs. S3 and S4. Note that there are two sets of genelets, ***v***, for each arraylet *y_m_*.

**Figures**

**
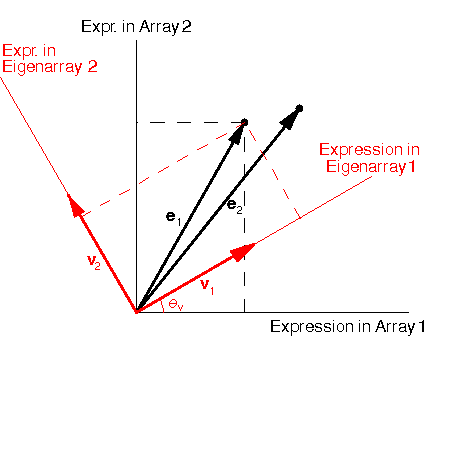
**

**Fig. S1.** The geometrical interpretation of a singular value decomposition of two genes expressed in two arrays. The expression vector of each gene, ***e***_1,2_, may be written as a sum of the ‘eigengene vectors’ ***v***_1,2_. The expression measured in the arrays and eigenarrays is indicated by dashed black and red lines, respectively. The angle of rotation between the two array coordinate systems, *θ_v_*, parameterizes the rotation matrix. Note that the eigengene characterised by the vector ***v***_m_ is only expressed in the *m^th^* eigenarray.

**
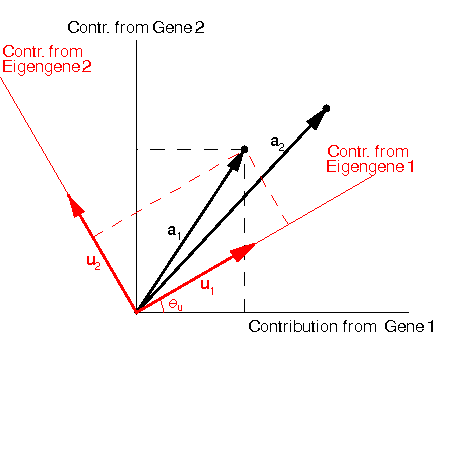
**

**Fig. S2.** The geometrical interpretation of a singular value decomposition of 2 genes expressed in 2 arrays (con't). Analogously to Fig. S1, the array-expression vector of each array, ***a****_1,2_*, may be written as a sum of the ‘eigenarray vectors’ ***u****_1,2_*. The contributions from genes and eigengenes are indicated through dashed black and red lines, respectively. The angle of rotation between the two gene coordinate systems, *θ_u_*, parameterizes the rotation matrix *u*. Note that the eigenarray characterized by the vector ***u****_m_* only receives a contribution from the *m^th^* eigenarray.

**
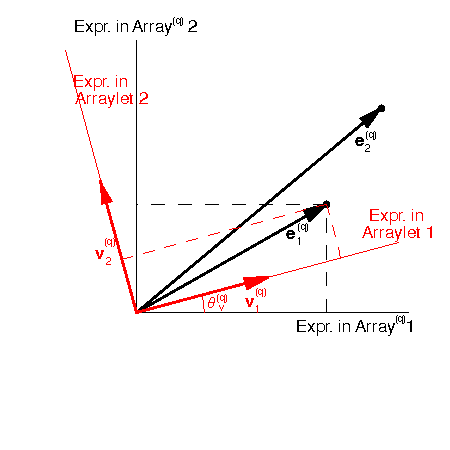
**

**Fig. S3.** The geometrical interpretation of a GSVD of two genes expressed in two datasets with two arrays each (viz. Fig. S1). A separate rotation (characterised by $\theta_{v}^{(p)}$and$\theta_{v}^{(q)}$) from axes indicating expression in arrays to axes indicating expression in arraylets is required. The genelets ***v***are only expressed in arraylet *m*. Only the plot showing the rotation for dataset *q* is shown.

**
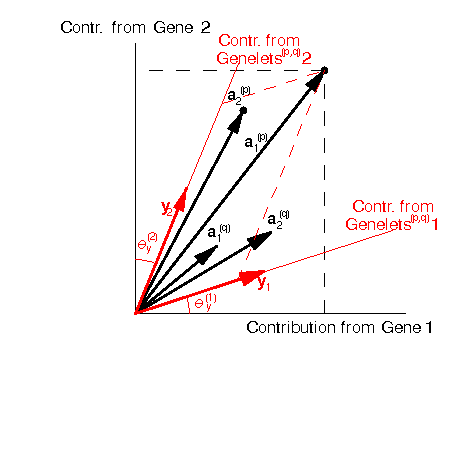
**

**Fig. S4.** The geometrical interpretation of a GSVD of two genes expressed in two datasets with two arrays each (viz. Fig. S2). Each of the four arrays receives contributions from the two genes. However, the transformed coordinate system is no longer orthogonal, with the rotation of the *n^th^* of axis determined by the *n^th^* column of the matrix *y*. The contribution from the two genes (genelets) to the first array of dataset (*p*) is indicated by dashed black (red) lines.
