# Supplementary material for: Combining transcriptional datasets using the generalized singular value decomposition
Source: BMC Bioinformatics. 2008 Aug 8;9:335. doi: 10.1186/1471-2105-9-335 (PMC2562393; doi:10.1186/1471-2105-9-335)
Supplement: Supplementary file 2 [file 1471-2105-9-335-S1.doc]

## Sheet: Additional File 1

Q-PCR fragment name	Full gene name	Barley1 genechip contig number 	First leaf tip	First leaf base	Root tip	Root m/zone	Floral early	Floral anthesis	Developing grain early	Developing grain mid	Coleoptile 3 day	Stem	scut 1day
HvAXA1	Arabinoxylan arabinofuranosidase 1	Contig12712	242985.3566367501	16244.718053329447	7749.022607513173	36324.243124566674	76629.57577290256	21013.599753531973	8783.278758542141	9792.843691148775	14173.33980425463	51233.77501352082	49479.41038839494
HvAXA2	Arabinoxylan arabinofuranosidase 2	Contig3596	1746024.2481498977	63250.40069940259	94160.07989121196	2911179.801247978	104201.17594992729	990519.783460235	413745.72892938496	81920.90395480226	372648.224540969	1863612.087614927	65752.2227421619
HvAXA3	Arabinoxylan arabinofuranosidase 3	Contig5239	649.5040151157298	203.99242313856914	152.98317185109636	566.2121562283337	6916.60871214516	82104.66088640464	317151.01793849654	4119.585687382298	13049.017228827955	8095.59221200649	400.67852129153016
*HvBNuc	Nuclease 1	Contig4111	11163.366336633664	3.021969719863407	315.4660368280628	6398.061737257717	819.1321152494729	13405.339230708476	647.4175035868006	536.5526492287056	146.5465975702999	849.3818854595912	170.03188097768333
*HvCesA1	Cellulose synthase 1	Contig3478	171067.54841757205	200356.9867404925	1125411.1422743497	833007.8576380865	241496.4911171524	179455.12961577394	271404.2924259681	136864.40677966102	1644255.7092399364	2306564.359113034	562871.431913898
*HvCesA2	Cellulose synthase 2	Contig3276	149208.78601794993	166851.2312399825	872656.3827978922	831413.2193205454	202386.67256749066	135497.99744729546	150508.07944191343	184039.54802259887	870533.042141875	1271048.5397512168	537830.486663547
HvCesA3	Cellulose synthase 3	Contig7166	527928.6726499763	56119.77269415707	1728.497365289818	8002.07996302288	116475.94360498198	62563.267461819465	105697.43023917996	127636.53483992467	45775.70169052819	49249.59437533802	180129.85493682732
*HvCesA4	Cellulose synthase 4	Contig9658	297.578845574063	1781.2909806207197	18405.78786333503	497209.3829443032	7009.862805841815	5579.639980634655	511.6386674259681	141.24293785310735	2337.620318692874	736313.5478637101	365.58259241927937
HvCesA8	Cellulose synthase 8	Contig20165	1913.0845536136042	3600.830540579921	21793.727689954103	878934.5967182806	24295.062274767653	10396.769508384314	977.8972095671982	1129.9435028248588	3114.1308743832406	1548979.1779340184	298.31539541413196
*Cont5737	Contig_5737	Contig5737	1547.0004723665566	938.000874253242	2573.33099609043	1923.965796163624	905.671113359044	1397.3856784472514	889.8063781321184	753.2956685499058	1059.6133624524791	15525.283937263384	1357.042583060365
HvCslA1	Cellulose synthase like A1	Contig10369	59.045819555975434	17091.650881538684	10464.473907870133	2831.060781141669	14685.148890434342	17059.108313894634	20047.337699316628	3483.9924670433143	58133.13920569441	1581.9361817198485	2538.605521759476
*HvCslA2	Cellulose synthase like A2	Contig12560	39253.660840812474	147604.9103890427	117218.04351521331	27987.05800785764	46367.83207940823	27260.02376655957	34835.91970387244	9675.141242937852	180243.46841381543	25550.97349918875	178749.41506785213
HvCslA4	Cellulose synthase like A4	Contig15434	66.00660066006601	1412.0153516061769	18917.027511700126	870.4235463029432	3621.3106113843987	187.80331551834595	533.0882352941177	1609.6579476861166	24288.505649477527	4196.451097468674	1293.836344314559
*HvCslA6	Cellulose synthase like A6	Contig18926	33.00330033003301	5.288447009760962	2.186939596728338	35.89375448671931	10.980323260716794	55.28789195166259	1781.7431850789098	8802.81690140845	8.495454931611588	8.409721638213774	207.22635494155156
HvCslA7	Cellulose synthase like A7	rbags19k14	10073.216816249409	5846.568556025061	12324.70678225395	6072.3364917957015	4122.147056964026	5533.427225914353	6807.018792710706	4865.034526051475	10824.638032839926	8501.216873985937	4910.50538137576
*HvCslC1	Cellulose synthase like C1	Contig18825	5810.108644307983	22022.074894361067	53090.472548019716	19124.10446036515	18410.570904722765	9114.91571673782	12106.705580865604	2448.210922787194	149648.1436544528	3195.420948260321	30276.087973795038
*HvCslC4	Cellulose synthase like C4	Contig8486	3070.382616910723	11529.214629170916	15594.722080571137	2580.6948617209773	12181.513561358031	16273.491483649486	13639.841970387244	1883.2391713747645	146871.71398527865	11310.167658193617	15603.065044454843
*HvCslD1	Cellulose synthase like D1	rbasd15h01	34655.95969138719	39523.53198309777	61680.90260071392	62913.103767044144	43053.3603085288	36590.59900532547	38639.84197038724	57085.68738229755	38872.03753134353	67042.99621416982	110037.43565746373
HvCslE1	Cellulose synthase like E1	Contig8067	240724.9646066709	2036.682208946525	84893.8679245283	1298369.4823203143	285263.7036100398	3837.1550547951233	35750.00889806378	16187.853107344634	90.06713580846073	14198.147647376962	4554.486429574169
HvCslE2	Cellulose synthase like E2	Contig18520	94.4733112895607	74.67579775608334	78.61635220125785	173.33025190663278	41.09502434089903	35.20971788213547	64.95586560364464	627.7463904582548	90.99733074496481	43.94267171444024	81.89050070191858
HvCslF6	Cellulose synthase like F6	Contig5706	4046.6068335695163	119588.73670406528	448219.4458609553	185902.47284492722	140105.58260099893	106700.84943444391	215531.57033029612	200164.78342749528	1038176.413491871	1648702.0010816657	316588.67571361724
HvCslH1	Cellulose synthase like H1	Contig7068	1804.4402456306093	14.570887367040653	51.20686724460309	206.262999768893	1298.2866536005563	82.9628977597817	202.875854214123	4852.47959824231	88.36851896788805	313.0070308274743	91.24941506785213
HvCslJ1	Cellulose synthase like J1	Contig20783	1122.1122112211222	17.37632588921459	392.0089227135546	529.4328786791098	41.72522839072382	26.327567596029805	22.865853658536587	268.2763246143528	93.45000424772746	16.81944327642755	9444.739638682253
HvDhn8	Dehydrin8	Contig2855	381740.92409240926	14739.657308633768	184765.04978932478	274084.7092605886	102119.20238931834	27268.11994839797	34100.16140602583	55482.897384305834	601747.939852179	302352.6196282903	12917.10945802338
HvEndogluI	(1,3;1,4)-beta-D-glucanase EI	HU14A02u	2242670.4455991182	116.02527709095331	25535.441101478835	2371672.059163393	13200.98628058418	303079.75001100305	278.5093963553531	97387.00564971751	424.65421014316917	51101.9469983775	2105773.280299485
HvEndogluII	(1,3;1,4)-beta-D-glucanase EII	Contig2834	1716.265155093686	359.4152217203361	127.4859765425803	847.3923426546492	227489.41012834292	1486.5102768364068	750.1067767653758	2730.6967984934086	780.5548815012538	101.40616549486208	127594.17407580721
HvGlyT3	Glycosyl transferase 3	Contig15389	363427.01936702884	624256.884744281	1746928.6503484615	766027.2706263	332036.7326294493	435711.4563619559	528097.4160022779	438017.89077212807	737988.3523416647	825537.4526771228	416708.58680393075
HvGlyT4	Glycosyl transferase 4	Contig8844	446843.0168477405	62997.23153140026	77892.86928437871	212861.10469147217	63871.151292912684	91801.63725188152	91837.80609339407	74034.83992467044	70907.14227938202	113896.0248783126	168051.00608329434
HvGlyT5	Glycosyl transferase 5	Contig18015	220.43772634230828	338.7731312836952	580.0611932687403	1455.9741160157153	1106.4045014857431	476.43149509264555	2730.8157744874716	5720.338983050848	1187.0096254954299	223.09356408869658	807.2063640617688
HvGSL1	Glucan synthase like 1	Contig8428	148665.56447803497	45572.27160134052	31418.918918918916	22209.382944303215	74854.58683694758	47839.003564983934	29299.544419134396	37641.24293785311	34716.4927606568	31767.171444023796	47987.83341132429
HvGSL2	Glucan synthase like 2	Contig4949	45748.70099196977	53320.340958764384	4265.468298487166	15929.050150219553	50150.68175591662	33433.82773645526	20411.268507972665	16760.828625235405	21372.23974763407	11759.734991887506	49374.122601778196
HvGSL3	Glucan synthase like 3	Contig19065	66485.59282002834	1665.9381222983145	6869.3693693693685	14952.623064478854	16046.026427261806	19661.32652612121	5980.3886674259675	3130.885122410546	10260.454582221144	6115.918514512348	5393.074403369209
HvGSL4	Glucan synthase like 4	Contig13152	80479.45205479451	39303.14731167128	23666.709161992178	31815.73068330637	93889.48599608016	35236.12517054707	31426.18166287016	36629.00188323917	40720.294426919034	51014.061654948615	39131.96069255966
HvHSP70	Heat shock protein70	Contig326	1508070.2734466407	616760.1631939384	2073157.8276389595	148116.47792928127	429566.28943541757	363714.18511509174	436857.5597949886	587382.2975517891	252586.34635606245	1199421.9848566793	1889790.0093589143
HvIRIP1	Ice recrystalisation inhibition protein1	HVSMEa0004N04r2	153.51913084553613	4.189130118024187	0.4249532551419343	2.3110700254217704	3.635329076310299	2.5306984727784867	2.0465546697038723	9.416195856873824	6.4709212974197206	7.774472687939427	1555.919513336453
HvIRIP3	Ice recrystalisation inhibition protein2	Contig7221	11868.209730751063	8879.134489290398	173.16845147033825	756.8754333256298	467.8510463425428	322.3889793583029	93.42966970387243	2518.8323917137477	1209.2534174553102	371.8226068144943	31565.863359850257
HvIsoamylase	Isoamylase	Contig7560	2751.5351913084555	2917.8201952498907	1155.8728539860615	1132.4243124566674	7559.377041579734	6893.7693470064405	25436.598329536828	31826.741996233523	7364.717301625819	3539.0751757706867	58.493214787084696
HvLimit-Dextrinase Inhibitor	Limit dextrinase Inhibitor	Contig6061	11.809163911195087	7.2854436835203265	5.311915689274179	17.333025190663278	101.15698299298222	78.12156155098808	328.3385535307517	3217231.638418079	24.265954865323952	13.52082206598161	38.020589611605054
HvLimit Dextrinase	Limit dextrinase	Contig11648	2385.4511100614077	12991.767448637622	3972.2505524392313	202.2186272244049	41992.79256496175	8042.48639291111	12539.151480637813	53170.11927181419	15857.801504489202	5611.141157382369	596.6307908282639
HvTrxh1	Thioredoxin 1	Contig171	1626700.5196032121	903628.1509543931	1170395.6314805371	1292535.2438178877	986288.487070873	753143.5676246644	693138.7030182232	578507.5329566855	1147075.9524387284	782341.806381828	872560.8329433786
HvTrxh2	Thioredoxin 2	Contig2980	1472024.0906943788	832161.5911409005	402227.81744008156	686202.9119482321	607920.2756527786	512807.53487962676	332574.0318906606	345550.8474576271	561950.9827711721	774479.4483504597	718838.7147090938
HvTrxh3	Thioredoxin 3	Contig318	165198.0198019802	31885.558006708772	68603.74841446879	119750.5384063173	74616.42070742561	35200.61606508175	31789.365136298424	70573.44064386317	86435.59029252683	91579.06539960194	146410.73326248673
HvTrxh4	Thioredoxin 4	Contig4154	280664.46228940325	43840.15736558356	58805.031446540874	213253.98659579386	69701.90301574254	23383.653888473218	32605.175113895217	103272.12806026365	86655.74698697728	97545.97079502433	15623.537669630323
HvTrxh5	Thioredoxin 5	Contig12146	808.5808580858086	1279.804176362153	208.8527314875563	35.89375448671931	349.1742796907941	467.97251401942975	716.9117647058824	125.75452716297787	8.495454931611588	4101.841729038769	313.49628055260365
HvUGE1	UDP-glucose epimerase 1	HVSMEm0008B04r2	58171.941426547004	187.6001748506484	3576.689897444614	18193.898775132886	12241.575520010116	2839.883807930989	1465.511104783599	3201.5065913370995	402.41041818328887	1906.4359113034072	2196.4202152550306
HvUGE2	UDP-glucose epimerase 2	Contig10066	9600.850259801606	7866.4578172810725	7298.572157062723	7620.753408828288	5381.8676107985075	3702.521896043308	2927.4629840546695	2142.184557438795	6268.705006875354	4799.891833423472	2544.4548432381844
HvUGE3	UDP-glucose epimerase 3	Contig9721	3613.604156825697	9.106804604400407	11.686214516403195	121.33117633464295	128.02680660049313	576.5591303199683	363.93080865603645	188.32391713747646	18.199466148992965	47.32287723093564	4158.867571361722
HvUXE1	UDP-xylose epimerase 1	Contig5788	5786.490316485593	14736.63121084074	21689.61414244433	19511.208689623298	11569.829929822343	8788.12552264425	8337.48576309795	15442.561205273068	17681.792445199386	27988.101676581933	2729.6833567306194
HvUXE2	UDP-xylose epimerase 2	Contig10276	26074.633915918752	7460.294331924814	23767.635560088387	34442.64694553579	13243.661882784345	5197.834602350249	1978.9293849658313	2424.670433145009	18541.211680012944	8301.78474851271	14991.810949929808
HvUXE3	UDP-xylose epimerase 3	Contig9821	36490.31648559282	12969.91111758706	25081.80350161482	26889.2997457823	13389.075045836757	11998.811672021478	7528.651765375854	13276.83615819209	18476.502467038747	67205.2460789616	29995.320542817033
HvUXS1	UDP Xylose synthase 1	Contig2031	983337.2697213037	1010771.5284860848	8059461.584225735	5391466.37393113	755579.4398432067	743912.019717442	1025892.4757972665	804919.9623352166	2417376.4458464775	14260951.865873445	417764.38933083764
HvUXS2	UDP Xylose synthase 2	Contig2918	118579.75122028026	61157.65700131138	84605.00594934556	114721.51606193668	33500.34772712904	28389.302114050144	240644.5757403189	72151.60075329567	254628.05683625877	262283.6668469443	15212.135392294495
HvUXS3	UDP Xylose synthase 3	Contig2916	3826.1691072272083	36.42721841760163	41880.20567737549	23809.79893690779	3871.362036627257	7630.606047269046	21283.27875854214	30320.15065913371	4956.321281242417	14514.60248783126	374.3565746373421
HvUXS4	UDP Xylose synthase 4	Contig2915	72945.20547945205	27852.251202098207	59173.678395376504	143286.34157614977	58601.50471012202	40188.59205140619	74375.94912680333	40842.74952919021	239183.45061878185	268248.6028483865	10713.032288254562
HvXTHA	Xyloglucan transglycosylase/hydrolase A	Contig2670	350495.9848842702	209446.79197629803	6181.653351464672	2996.6874662968958	167342.10027185938	51902.425069319135	26006.371013667424	20833.333333333332	2480975.491385586	832020.6868577609	13309.156137888005
HvXTHB	Xyloglucan transglycosylase/hydrolase B	Contig2671	6156.510785703039	3191086.259653213	1342221.2306646267	11324.243124566676	385929.6958968199	150391.70811143875	99515.94533029612	159259.25925925924	13394734.287794225	2278048.945375879	1751649.5086569958
HvXTHC	Xyloglucan transglycosylase/hydrolase C	Contig2958	13096.362777515353	122655.30137452036	296863.8449770525	14114.860180263462	48651.76708604665	42748.998723647725	89634.64550113895	136864.40677966102	684720.5370864677	284738.37209302327	162377.1642489471
HvXTHD	Xyloglucan transglycosylase/hydrolase D	HE01I24u	246551.72413793104	348621.2297828938	526790.1155872854	6105182.574532009	92043.37105645823	240695.8320496457	138086.38240318905	23132.45448838669	1431626.627841139	9746684.018388318	54936.82732802995
HvXTHE	Xyloglucan transglycosylase/hydrolase E	Contig5258	45819.58195819582	51043.08321497285	60658.41461458834	35.89375448671931	23079.17545092527	25864.20240633968	27643.472022955524	13916.83433936955	1255420.1002463682	225189.9195469963	52053.66631243358
HvCesA5	Cellulose synthase 5		86.60053534876397	13.660206906600612	24.434812170661225	705.4541252599954	17.280984594634464	16.064433783724308	4.5380125284738035	84.7457627118644	11.32411227048451	1912.8583017847484	7.311651848385587
HvCesA6	Cellulose synthase 6		125476.3029444182	84999.27145563165	524628.1659017508	301970.1871966721	119722.450527913	83467.93715065358	104666.14464692482	133498.11676082862	593076.1142117609	579340.18388318	185049.13430042114
HvCesA7	Cellulose synthase 7		1169.1072272083136	1041.8184467434066	3598.291687914329	86919.34365611279	4362.394891572359	3187.5797720170767	264.27249430523915	1506.5913370998117	396.3439294669579	189872.90427257976	391.9045390734675
HvCslA8	Cellulose synthase like A8		7799.7799779978	4625.880148680911	12083.38800682325	5096.913137114142	9787.860154602951	1696.811731564121	2010.8500717360116	1131.7907444668008	1554.6682524849205	9955.007989235555	4667.906482465462
HvCslC2	Cellulose synthase like C2		75862.06896551725	67466.85123123998	12880.33316335203	18638.77975502658	132371.81513561358	33560.362660094186	31516.941913439634	18989.32831136221	38394.807085658824	93540.42725797728	85040.3603182031
HvCslC3	Cellulose synthase like C3		5899.858290033066	24.284812278401088	128372.32279449259	259.4176103535937	78.71277739141429	14.524008626380882	3.025341685649203	534.3691148775895	4247.553182884413	11.154678204434829	12002.22274216191
HvCslD2	Cellulose synthase like D2		1393.4813415210203	1741.221040361358	51070.88220295767	2363.0691009937605	829.8033761143073	730.601646054311	515.1978929384966	9.416195856873824	24.265954865323952	128.44780962682532	2845.6948993916703
HvCslF3	Cellulose synthase like F3		47.23665564478035	2484.336296080431	217719.48835628078	4783.914952623065	284.50401466776253	259.6716693807491	27.58399772209567	94.16195856873823	1275881.6630267734	642.2390481341265	32.171268132896586
HvELF1	Elongation Factor alpha		85309.40009447331	1589281.2909806208	1471012.8760836306	48307.141206378554	494578.61794271984	1301122.309757493	2358660.4854783597	1544915.2542372881	484439.45644261106	247397.24175229852	130436.94431445953
HvGlyT1	Glycosyl transferase 1		4.7236655644780345	9.106804604400407	6632.457929627741	444.8809798936908	3.1611557185306944	0.44012147352669334	0.8898063781321184	47.080979284369114	6.066488716330988	37.18226068144943	1272.2274216190922
HvGlyT2	Glycosyl transferase 2		708.5498346717053	257.4190101510515	9679.37276899541	3478.1603882597647	344.56597331984574	194.7537520355618	89.87044419134396	753.2956685499058	64.7092129741972	2105.868036776636	3296.0926532522226
HvGlyT6	Glycosyl transferase 6		286.02860286028607	95.19204617569733	37.90695300995786	403.8047379755922	162.50878425860856	72.40081088908197	448.94787183165954	6690.140845070422	144.42273383739698	308.35646006783844	12029.755579171095
HvGSL6	Glucan synthase like 6		12061.092741300583	27872.286172227887	26333.29083800782	7609.198058701179	82855.47196054878	39470.09374587386	20892.65375854214	19609.227871939736	32546.71196311575	7781.233098972417	56580.48666354703
HvGSL7	Glucan synthase like 7		16773.927392739275	8534.042488894262	12448.789164443277	7322.325915290739	24356.553056921995	15653.71350340942	10748.445719751317	4200.201207243461	13323.705151077507	13337.818518207047	15472.901168969183
HvGSL8	Glucan synthase like 8		47136.96369636964	25548.739334965197	15256.637361676068	11961.59368269921	36224.08643710471	32561.935602769663	20802.994978479197	30818.24279007378	18488.94174949735	13842.401816499872	50385.22848034007
HvMannanase	Mannanase		3250.825082508251	315.795835725726	253.68499322048723	5321.249102656137	1412.0695713281798	645.0254061027302	771.1621233859398	11670.020120724346	872.9079942230907	2121.3522832394247	1269.9256110520723
HvRD17	Responsive to dehydration 17		296897.6897689769	8871.495774279008	134533.2341920716	223088.6575735822	160895.4087608339	14917.857989100388	19339.13199426112	64520.45606975184	242483.64624925665	156738.2894626188	7391.073326248672
